# Spatial Gradient of Microstructural Changes in Normal-Appearing White Matter in Tracts Affected by White Matter Hyperintensities in Older Age

**DOI:** 10.3389/fneur.2019.00784

**Published:** 2019-07-25

**Authors:** Susana Muñoz Maniega, Rozanna Meijboom, Francesca M. Chappell, Maria del C. Valdés Hernández, John M. Starr, Mark E. Bastin, Ian J. Deary, Joanna M. Wardlaw

**Affiliations:** ^1^Neuroimaging Sciences, Centre for Clinical Brain Sciences, University of Edinburgh, Edinburgh, United Kingdom; ^2^UK Dementia Research Institute at the University of Edinburgh, Edinburgh, United Kingdom; ^3^Alzheimer Scotland Dementia Research Centre, University of Edinburgh, Edinburgh, United Kingdom; ^4^Department of Radiology and Nuclear Medicine, Erasmus MC–University Medical Centre Rotterdam, Rotterdam, Netherlands; ^5^Centre for Cognitive Ageing and Cognitive Epidemiology, University of Edinburgh, Edinburgh, United Kingdom; ^6^Department of Psychology, University of Edinburgh, Edinburgh, United Kingdom

**Keywords:** brain, aging, diffusion MRI, white matter hyperintensities, tractography, cerebral small vessel disease

## Abstract

**Background and Purpose:** White matter hyperintensities (WMH) are commonly seen on structural MRI of older adults and are a manifestation of underlying and adjacent tissue damage. WMH may contribute to cortical disconnection and cognitive dysfunction, but it is unclear how WMH affect intersecting or nearby white matter tract integrity. This study investigated the effects of WMH on tract microstructure by determining the spatial distribution of water diffusion characteristics in white matter tract areas adjacent to both intersecting and nearby WMH.

**Methods:** We used diffusion and structural MRI data from 52 representative participants from the Lothian Birth Cohort 1936 (72.2 ± 0.7 years) including a range of WMH burden. We segmented WMH, reconstructed 18 main white mater tracts using automated quantitative tractography and identified intersections between tracts and WMH. We measured mean diffusivity (MD) and fractional anisotropy (FA) in tract tissue at 2 mm incremental distances from tract-intersecting and non-intersecting (nearby) WMH.

**Results:** We observed a spatial gradient of FA and MD abnormalities for most white matter tracts which diminished with a similar distance pattern for tract-intersecting and nearby WMH. Overall, FA was higher, while MD was lower around nearby WMH compared with tract-intersecting WMH. However, for some tracts, FA was lower in areas immediately surrounding nearby WMH, although with faster normalization than in FA values surrounding tract-intersecting WMH.

**Conclusion:** WMH have similar effects on tract infrastructure, whether they be intersecting or nearby. However, the observed differences in tract water diffusion properties around WMH suggest that degenerative processes in small vessel disease may propagate further along the tract for intersecting WMH, while in some areas of the brain there is a larger and more localized accumulation of axonal damage in tract tissue nearby a non-connected WMH. Longitudinal studies should address differential effects of intersecting vs. nearby WMH progression and how they contribute to cognitive aging.

## Introduction

White matter hyperintensities (WMH), or leukoaraiosis, are routinely found in brain magnetic resonance imaging (MRI) scans of older people and have been described as white mater (WM) degeneration characterized by axonal loss, demyelination and gliosis, on neuropathological examination (1). The presence of WMH contributes to cortical “disconnection” (2, 3) and cognitive and functional decline (4–6). Several studies have observed associations between WMH volume within specific WM tracts and cognition (7, 8), suggesting that the presence of WMH affects the performance of WM pathways. The influence of WMH on WM tracts can be explored further by assessing, not just the extent of the observable damage in the tract, but also the invisible changes in tract tissue close to WMH.

Diffusion MRI (dMRI) allows the assessment of microstructural quality of WM *in-vivo* through the tissue diffusion properties. Fractional anisotropy (FA) is an indicator of the degree of directionality of the water diffusion within the tissue, while mean diffusivity (MD) reflects the degree of diffusion in all directions. These parameters can therefore be used to assess changes in structural barriers within WM, such as axonal membranes or myelin (9). Histology studies of WM have shown that reduced axonal density, and anomalies in the myelin sheaths underlie abnormalities observed in these water diffusion parameters (10, 11).

Previous dMRI studies of whole-brain normal-appearing white matter (NAWM) have observed gradual changes of FA and MD with distance from WMH and described them as a “penumbra” effect of WMH on NAWM (12, 13). A similar distance pattern of NAWM tissue damage has been observed within the corticospinal tract, caused by the WMH that crossed the tract, as well as by nearby WMH, outside the tract (14).

In the current study, we used dMRI tractography (15) to reconstruct 18 of the main WM pathways of the brain. We measured FA and MD within these tracts to investigate how the presence of WMH affects the integrity of surrounding NAWM in tracts segmented in a group of older age subjects.

## Methods

### Participants

The LBC1936 comprises a group of community-dwelling individuals born in 1936, most of whom took part in the Scottish Mental Survey of 1947. At ~70 years of age, the LBC1936 participants were recruited for follow-up cognitive and other medical and psycho-social assessments (16, 17). During a second wave of this longitudinal study, at ~73 years of age, 700 participants underwent comprehensive MRI to assess brain structure (18). Written informed consent was obtained from all participants under protocols approved by the National Health Service Ethics Committees.

The current study used imaging data from the second wave of the LBC1936. A sample was chosen with three requirements: it represented all levels of WMH burden, each participant had available structural and diffusion MRI data, and participants did not have a history of stroke (self-reported). We selected the participants in recruitment order, blind to any other medical or imaging data. In order to create a sample representative of all levels of WMH burden, participants were selected based on Fazekas score (19), as we have previously shown that, there is a strong correlation between Fazekas score and WMH volume (20). A sample of 60 participants, with 10 participants per Fazekas total score of 1–6 (sum of deep + periventricular 0–3 scores) was intended. However, only eight participants with a Fazekas score of 6 were selected, as all other participants with this score had a history of stroke. We completed the sample with an additional case for Fazekas scores 2 and 3, as these were the most frequent scores observed in the LBC1936. The final sample therefore consisted of ten participants per Fazekas scores 1, 4 and 5; 11 participants per Fazekas scores 2 and 3; and eight participants with a Fazekas score of 6.

### Imaging Acquisition

All MRI data were acquired using the same GE Signa Horizon HDxt 1.5 T clinical scanner (General Electric, Milwaukee, WI, USA), with a self-shielding gradient set at a maximum of 33 mT/m and an 8-channel phased-array head coil. The full details of the imaging protocol can be found in Wardlaw et al. (18). Briefly, the MRI examination comprised a high-resolution 3D T1-weighted (T1W), T2W, T2^*^-weighted (T2^*^W) and FLAIR structural scans, as well as dMRI. The dMRI protocol consisted of seven T2W volumes (*b* = 0 s/mm^2^) and sets of diffusion-weighted (*b* = 1,000 s/mm^2^) single-shot, spin-echo, echo-planar (EP) volumes acquired with diffusion gradients applied in 64 non-collinear directions (21). All sequences, except for the T1W, were acquired in the axial plane with a field-of-view (FOV) of 256 × 256 mm, contiguous slice locations, and image matrices and slice thicknesses designed to give 2 mm isotropic voxels for dMRI, and voxel dimensions of 1 × 1 × 2 mm for T2W and T2^*^W, and 1 × 1 × 4 mm for FLAIR. The high-resolution 3D T1W scan was acquired in the coronal plane with a FOV of 256 × 256 mm and voxel dimensions of 1 × 1 × 1.3 mm.

### Visual Scoring of White Matter Hyperintensities

WMH were defined according to the STRIVE criteria (22). All assessments used validated visual or computational methods and were performed blind to all patient demographic, clinical and tractography characteristics. A qualitative assessment of WMH load was performed by an expert neuroradiologist who scored hyperintensities on the FLAIR and T2W scans using the Fazekas scale, after training on a standard data set. A second consultant neuroradiologist cross-checked a random sample of 10% of ratings, all scans with stroke lesions, and any scans where the first rater was uncertain. The final measurements were those agreed as discussed amongst the two neuroradiologists. A total score ranging from 0 to 6 was obtained by summing the periventricular and deep WMH Fazekas scores. The Fazekas scale is one of the most widely used visual rating scales and has been in use for over two decades (19).

### Whole Brain WMH and NAWM Segmentation

All structural MRI volumes were registered to the corresponding T2W volume using rigid body registration (23). Whole brain NAWM and WMH tissue masks were obtained using the multispectral coloring modulation and variance identification (MCMxxxVI) method (24). In brief, T2^*^W and FLAIR volumes were mapped into red-green color space and fused; the minimum variance quantization clustering technique was then used in the resulting image to reduce the number of color levels, thereby allowing WMH to be separated from other tissues in a reproducible and semiautomatic manner. The same method was used to extract the NAWM from the T1W and T2W volumes. Any silent stroke lesions were identified by a neuroradiologist and excluded from the masks manually by a trained image analyst.

### Diffusion Tensor Imaging Analysis and Tractography

**Figure 1:**
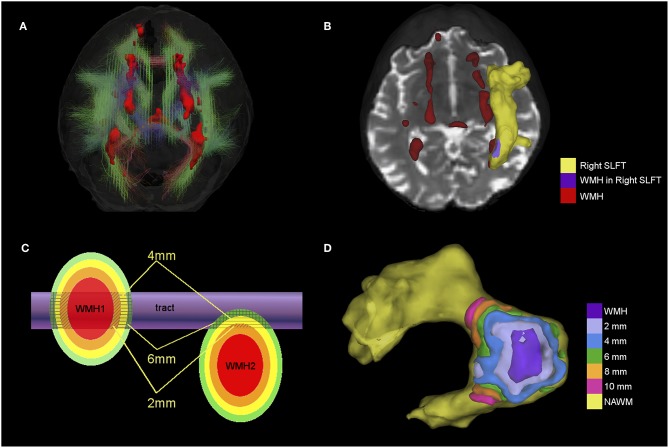
**(A)** 3D view of 18 tracts (streamlines) and WMH (red surface) from a participant with a total Fazekas score of 3. Streamlines are color-coded according to the main direction of the middle segment of the tract (red = x axis, green = y axis and blue = z axis). **(B)** Example showing the superior longitudinal fasciculus temporal ending (SLFt) as a yellow surface and the area where the SLFt intersects with WMH in purple. Remaining WMH are shown in red. **(C)** Schematic figure of the spatial analysis of the effects of tract-WMH (WMH1) and the nearby-WMH (WMH2) showing 2, 4, and 6 mm contours around the WMH. Average FA and MD are measured only where the contours intersect with tract-NAWM (patterned areas). **(D)** Example of the spatial analysis contours for tract-WMH in the SLFt; the tract-WMH area (purple) is dilated by 2 mm at a time to create surface contours within the tract-NAWM at different distances from the WMH edge.

dMRI volumes were pre-processed using FSL 4.1 (25). First, brain extraction was performed using BET (26), and second, bulk motion and eddy current induced distortions were removed by registering all volumes to the first T2W EP volume (23). Third, DTIFIT was used to obtain the water diffusion tensor on a voxel-wise level, and to calculate parametric maps of FA and MD from the diffusion tensor eigenvalues. This was followed by automatic tractography using Tracula implemented in Freesurfer5.3 [TRActs Constrained by UnderLying Anatomy; (27)]. Tracula uses global probabilistic tractography (28) and anatomical priors of the white matter pathways derived from a set of training subjects; its accuracy has been evaluated against manual tract labels (27). Registration to the tract atlas containing the priors was performed by affine registration to the MNI125 template (23). We reconstructed the 18 white matter pathways included in Tracula (corpus callosum: forceps major and forceps minor, and bilateral corticospinal tract (CST), inferior longitudinal fasciculus (ILF), uncinate fasciculus (UNC), anterior thalamic radiation (ATR), cingulum: cingulate gyrus (CCG) and angular bundle (CAB), and superior longitudinal fasciculus: parietal (SLFp) and temporal (SLFt) segments). All white matter tracts were visually inspected and those not following the expected paths were discarded from further analysis. See Figure 1A for an example showing the 18 tracts and WMH in a representative participant. The tract masks were binarized after applying a threshold of 1% to the tract posterior probability.

### WM Tract-WMH and WM Tract-NAWM Intersections

To obtain the areas of the tracts that intersected with WMH for each individual, we first non-linearly registered the T2W volume to the averaged T2W EP volume (S0) using RNiftyReg (29, 30). This registration was then applied to the whole-brain WMH and NAWM masks created previously in order to overlap them with the Tracula tracts-masks in diffusion space and obtain the intersections. This way the tracts were divided into *tract-WMH* (as the intersection between the tract and the whole-brain WMH mask) and *tract-NAWM* (as the intersection between the tract and the whole-brain NAWM), see example in Figure 1B. These were subsequently overlaid onto the FA and MD parametric maps for quantitative measurements. Averages of FA and MD values from each tract area (WMH and NAWM) were obtained. Please note that not all tracts had a tract-WMH intersection, and the area of WMH overlap varied for different tracts and between participants. The percentages of overlap were quantified as the tract-WMH percentage volume (% WMHvol) for each individual WM tract and for all WM tracts combined. These were calculated by dividing the tract-WMH volume by the total WM tract volume (tract-WMH plus tract-NAWM).

### Spatial Analysis of Tracts

#### Spatial Contours of Tract-WMH

We assessed how the intersection or proximity of the WMH was associated with changes in tract integrity by creating approximately equidistant 3D contours around the tract-WMH, which propagated into the tract-NAWM for each tract. To achieve this, we dilated the tract-WMH masks in 3D by 2 mm increments (1 voxel in dMRI-space) up to 10 mm, and then subtracted from each dilated ROI the previous ones. That is, the tract-WMH mask was subtracted from the 2 mm ROI to obtain a contour at about 2 mm from the WMH edge; the tract-WMH and 2 mm ROI were subtracted from the 4 mm ROI to obtain a contour at about 4 mm from the WMH edge, and so on (the distances quoted are approximate as they are limited by the finite voxel size). For each contour, only the voxels overlapping with the tract-NAWM were kept for each tract, so no other tissues were included. See “WMH1” in Figure 1C for a graphical representation of this approach and Figure 1D for an example of contours in the SLFt. Means of FA and MD were obtained for each contour for parametric assessment of the effects of tract-WMH.

#### Nearby-WMH

The spatial analysis was repeated for WMH that were nearby, but that did not intersect the tract, to assess their association with changes in the WM tract. We considered only those nearby-WMH for which any of the 2–10 mm contours intersected with the tract-NAWM (see “WMH2” in Figure 1C). Any voxel already belonging to a WMH-tract contour was automatically excluded from the nearby-WMH contours, to ensure the areas measured were not connected to an intersecting WMH. Means of FA and MD were obtained for each nearby-WMH contour that intersected with an individual tract-NAWM.

### Statistical Analyses

All statistical analyses were performed in R v.3.5 with packages *car* (31) and *lmerTest* (32). Plots were made with *ggplot2* (33). Analyses were performed including all tracts within a model, and also for each individual tract.

We are aware of the problem of multiple comparison, and so, for transparency, we report all *p*-values and coefficients estimated from the models. Methods for correcting for multiple comparison, such as Bonferroni, can be overly conservative and difficult to interpret where variables are highly correlated (such as brain MRI biomarkers), and therefore we chose not to apply any correction here; however to provide a compromise between Type I and Type II errors, we interpret results as significant if *p* < 0.01.

#### Water Diffusion in Tract-NAWM Spatial Contours for Tract-WMH

We assessed the spatial changes of FA and MD values with distance from the tract-WMH with a repeated-measurements linear mixed model for each parameter, with the repeats being FA or MD obtained in tract-WMH, and tract-NAWM at 2, 4, 6, 8 and 10 mm from the tract-WMH. As the trajectories of FA and MD show an asymptotic relationship of water diffusion with distance (Figures 3A,B), log(distance+1) was used as fixed effect (with tract-WMH coded as 0 mm). The model including all tracts was a three-level model, with the measurements for each tract included in the model as repeats at each distance. A model with random intercept and slope for both participant and tract gave the best fit (lowest Bayesian information criterion) and residual distribution.

The analysis was repeated for each tract separately, with log(distance+1) as fixed effect and random intercept and slope for participant. Type III Wald F tests with Kenward-Roger df approximation were used to obtain F and *p*-values.

#### Water Diffusion in Tract-NAWM Spatial Contours for Nearby-WMH

FA and MD values for spatial distances at 2, 4, 6, 8, and 10 mm from nearby-WMH were measured for all tracts. We then assessed the effect of the two types of WMH (tract or nearby) on the water diffusion measurements in NAWM spatial contours. The value of WMH was not available for nearby-WMH, hence we compare the trajectories between tract-WMH and nearby-WMH for distances 2–10 mm only. We used a repeated-measure linear mixed model with distance and WMH type as fixed effects; the interaction of distance and WMH type was included as fixed effect only if it improved the model (lowest Bayesian information criterion). The best fitting model for both FA and MD included log(distance), WMH type (tract-WMH and nearby WMH), and random intercept and slope for both participant and tract.

The analysis was repeated for each tract separately, with log(distance) and WMH type as fixed effects and random intercept and slope for participant. The interaction term [log(distance):WMH type] was included in the model for those tracts that showed an improvement in model fit with this term. Type III Wald F tests with Kenward-Roger df approximation were used to obtain F and *p*-values.

## Results

### Participant Demographics

Data from 52 participants (27 male) with a mean age of 72.2 (standard deviation 0.7) years were used for analysis (Table 1). Out of the 60 participants in the initial sample, Tracula failed to produce viable tractography outputs in six participants, one participant's WMH were too small to be segmented, while another participant's WMH did not intersect with any of the tracts of interest, and they were therefore excluded from the analysis. One participant had a silent stroke lesion, which was excluded from the tissue segmentation masks.

**Table 1:** Percentage of WM tracts affected by WMH.

**WM tract**	**% *N* with WMH in tract**
Anterior thalamic radiation L	84.6
Anterior thalamic radiation R	82.7
Cingulate cingulum L	75.0
Cingulate cingulum R	78.9
Cingulum angular bundle L	46.2
Cingulum angular bundle R	50.0
Corticospinal tract L	94.2
Corticospinal tract R	92.3
Inferior longitudinal fasciculus L	78.9
Inferior longitudinal fasciculus R	76.9
Forceps Major	67.3
Forceps Minor	75.0
Superior longitudinal fasciculus, parietal L	76.9
Superior longitudinal fasciculus, parietal R	76.9
Superior longitudinal fasciculus, temporal L	78.9
Superior longitudinal fasciculus, temporal R	84.6
Uncinate fasciculus L	76.9
Uncinate fasciculus R	82.7

*WM, white matter; L, left; R, right; WMH, white matter hyperintensity*.

*% of N with a WMH tract, excluding participants without a WMH in that tract and those that were excluded after data quality assessment*.

Participants varied in Fazekas score, with seven participants with a total Fazekas score of 1; nine participants with a score of 2; 10 participants with a score of 3; nine participants with each scores of 4 and 5; and eight participants with a Fazekas score of 6. Consequently, there was also a variation in number of tracts affected by WMH, but most participants (*N* = 46) had at least 8 tracts intersecting WMH. The corticospinal tracts had the highest percentage of participants with WMH intersecting the tract, followed by the anterior thalamic radiations and right temporal superior longitudinal fasciculus (Table 1). The lowest percentage of participants with a WMH was observed for the bilateral cingulum angular bundle.

### Tract-WMH Percentage Volumes

**Figure 2:**
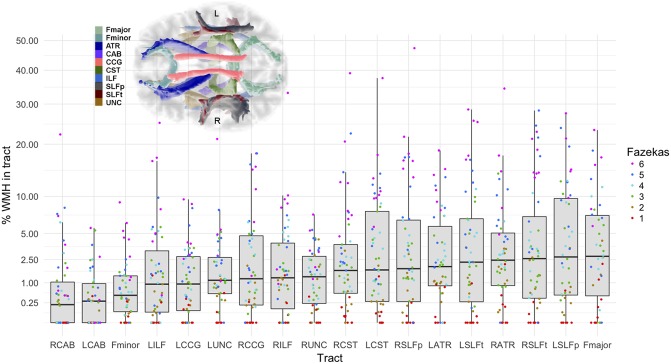
Percentage of the tract intersecting a WMH according to severity of WMH. Vertical axis is in square-root scale for easier visualization. Tracts are ordered by median % WMH overlap in tract (less affected on the left, more affected on the right of plot). The boxes represent the lower and upper quartiles and the median measurement (thick line) for each tract, while whiskers indicate the sample minimum and maximum, excluding outliers that differ from the lower and upper quartiles by more than 1.5 times the interquartile range. The dots represent individual data, color coded by total Fazekas score (1 = low, 6 = high WMH load). Inset shows location of the tracts on axial projection of the brain. L, left; R, right; Fmajor, forceps major; Fminor, forceps minor; ATR, anterior thalamic radiation; CCG, cingulate cingulum; CAB, cingulum angular bundle; CST, corticospinal tract; ILF, inferior longitudinal fasciculus; SLFp, parietal superior longitudinal fasciculus; SLFt, temporal superior longitudinal fasciculus; UNC, uncinate fasciculus.

Median tract-WMH volumes ranged from 0.2 to 2.8% of total WM tract volume, with maximum overlaps seen up to 47% (see Figure 2 and Supplementary Table 1). The forceps major of the corpus callosum, the superior longitudinal fasciculus segments and the anterior thalamic radiations presented the largest median overlap with WMH, while the cingulate angular bundles and forceps minor presented the lowest overlap.

### Water Diffusion in Tract-WMH and Tract-NAWM Spatial Contours

#### All Tracts

We observed some outliers in the model caused by CSF contamination in some NAWM contours. Contours with MD > 10^−3^ mm^2^/s were therefore excluded from analysis as we found in this population MD is unlikely to be over this value in white matter (13).

Results from the repeated-measurements linear mixed model including all WM tracts showed that FA increases significantly [estimate = 0.024, *F*_(1, 27.3)_ = 53.1, *p* < 0.001], while MD decreases significantly [estimate = −0.085, *F*_(1, 42.1)_ = 199.1, *p* < 0.001], and logarithmically, with the distance from the WMH (Table 2, top section).

**Table 2:** Results from the repeated-measures linear mixed model analysis for the full models including all tracts.

**Outcome variable**	**Predictor**	**Estimate**	**Std. error**	**Df.res**	***F***	***p***
**TRACT-WMH SPATIAL CONTOURS**						
FA	Log(distance+1)	0.024	0.003	27.3	53.1	<0.001
MD	Log(distance+1)	−0.085	0.006	42.1	199.1	<0.001
**TRACT-WMH vs. NEARBY-WMH**						
FA	Log(distance)	0.003	0.006	26.0	0.39	0.540
	WMH “nearby”	0.015	0.002	6513.9	81.1	<0.001
MD	Log(distance)	−0.027	0.003	33.2	91.5	<0.001
	WMH “nearby”	−0.012	0.001	6510.8	81.9	<0.001

*Top section shows the results for water diffusion in tract-WMH spatial contours; bottom section shows the results for the models including tract-WMH and nearby-WMH contours*.

*WMH, white matter hyperintensity; FA, fractional anisotropy; MD, mean diffusivity in (10^−3^ mm^2^/s)*.

**Figure 3:**
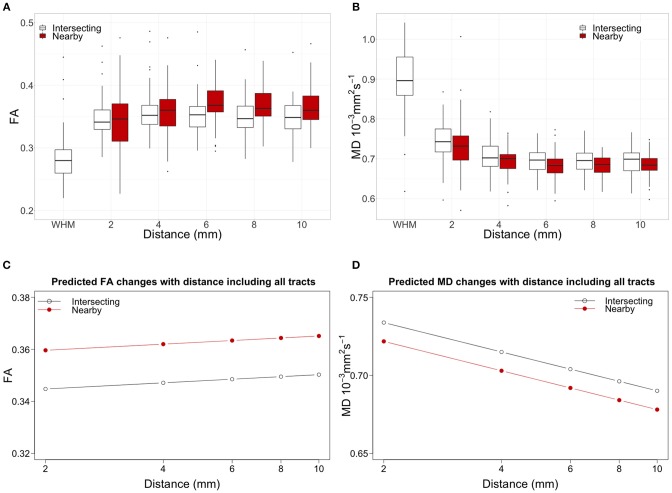
Top panel shows the water diffusion changes in tract-NAWM with distance to intersecting (tract-WMH) and nearby WMH. Boxplots of **(A)** fractional anisotropy (FA) and **(B)** mean diffusivity (MD) measurements averaged for all tracts. WMH measurements were only performed in tract-WMH. In all box plots, the boxes represent the lower and upper quartiles and the median measurement (thick line) for each group. Whiskers indicate the sample minimum and maximum, whereas the represented outliers (dots) differ from the lower and upper quartiles by more than 1.5 times the interquartile range. The bottom panel shows the model predicted water diffusion changes in tract-NAWM with distance from tract-WMH for **(C)** FA and **(D)** MD. The distance scale is logarithmic for easier visualization. Open circles show the predicted values for the WMH-tract contours, while red circles show the predicted values for the nearby-WMH contours.

Figures 3A,B show in white the boxplots for FA and MD measured in tract-WMH (intersecting) and each distance to tract-WMH, including results for all WM tracts. The open circles in Figures 3C,D show the models predicted water diffusion changes in tract-NAWM with distance from tract-WMH.

#### Individual Tracts

FA increases significantly with distance for all tracts except for the bilateral CAB and ILF, while MD decreases significantly with distance all tracts (Table 3). Supplementary Figure 1 shows plots of the changes for individual tracts for each participant.

**Table 3:** Results from the repeated-measures linear mixed model analysis for the analysis of water diffusion in tract-WMH spatial contours in individual tracts.

**Outcome**	**FA**					**MD**				
**Predictor**	**Log(distance+1)**					**Log(distance+1)**				
**Tract**	**Estimate**	**Std. error**	**Df.res**	***F***	***p***	**Estimate**	**Std. error**	**Df.res**	***F***	***p***
F Major	0.034	0.007	33	20.8	<0.001	−0.126	0.007	33	300.1	<0.001
F Minor	0.040	0.006	35	52.4	<0.001	−0.076	0.007	35	121.1	<0.001
L ATR	0.033	0.003	42	114.8	<0.001	−0.105	0.007	42	259.1	<0.001
L CAB	0.000	0.013	16	0.0	0.999	−0.044	0.013	16	12.2	0.003
L CCG	0.037	0.005	34	66.4	<0.001	−0.076	0.008	34	100.1	0.000
L CST	0.022	0.006	44	13.8	0.001	−0.059	0.005	44	170.7	<0.001
L ILF	0.010	0.005	39	3.9	0.057	−0.112	0.009	39	161.7	<0.001
L SLFp	0.017	0.004	38	16.5	<0.001	−0.078	0.006	38	164.5	<0.001
L SLFt	0.020	0.003	40	34.0	<0.001	−0.087	0.006	40	198.6	<0.001
L UNC	0.023	0.003	37	51.4	<0.001	−0.100	0.007	37	209.7	<0.001
R ATR	0.040	0.004	42	106.8	<0.001	−0.094	0.007	42	203.7	<0.001
R CAB	0.013	0.012	20	1.1	0.310	−0.059	0.019	20	10.1	0.005
R CCG	0.036	0.004	28	75.1	<0.001	−0.085	0.009	28	97.6	<0.001
R CST	0.034	0.005	47	54.5	<0.001	−0.070	0.006	47	142.9	<0.001
R ILF	−0.001	0.004	38	0.0	0.834	−0.114	0.007	38	240.7	<0.001
R SLFp	0.024	0.004	38	28.7	<0.001	−0.088	0.006	38	185.7	<0.001
R SLFt	0.027	0.004	42	46.3	<0.001	−0.084	0.005	42	250.1	<0.001
R UNC	0.033	0.004	37	60.2	<0.001	−0.083	0.009	37	86.2	<0.001

*FA, fractional anisotropy; MD, mean diffusivity in (10^−3^ mm^2^/s); L, left; R, right; F, forceps; ATR, anterior thalamic radiation; CCG, cingulate cingulum; CAB, cingulum angular bundle; CST, corticospinal tract; ILF, inferior longitudinal fasciculus; SLFp, parietal superior longitudinal fasciculus; SLFt, temporal superior longitudinal fasciculus; UNC, uncinate fasciculus*.

### Water Diffusion in Tract-NAWM Spatial Contours: Tract-WMH and Nearby-WMH Compared

#### All Tracts

Figures 3A,B show the changes of water diffusion parameters with distance to the intersecting WMH (white boxplots) compared with those of nearby WMH (red boxplots). For both types of WMH the parameters show similar trajectories, for FA and MD. Figures 3C,D show the predicted values obtained by fitted models for both FA and MD.

Table 2 bottom section shows the results from the models. For FA, the statistical model showed significant effects for WMH type [estimate = 0.015, *F*_(1, 6513.9)_ = 81.1, *p* < 0.001], with higher FA for nearby-WMH contours than for the same distance tract-WMH contours. The effect of distance was not significant [estimate = 0.003, *F*_(1, 26.0)_ = 0.39, *p* = 0.540].

For MD, we observed a significant effect of WMH type [estimate = −0.012, *F*_(1, 6510.8)_ = 81.9, *p* < 0.001], with higher MD for tract-WMH contours than for the equivalent nearby-WMH contours. The log(distance) effect was also significant [estimate = −0.027, *F*_(1, 38.1)_ = 91.5, *p* < 0.001], indicating a decrease of MD with distance from the WMH.

#### Individual Tracts

**Figure 4:**
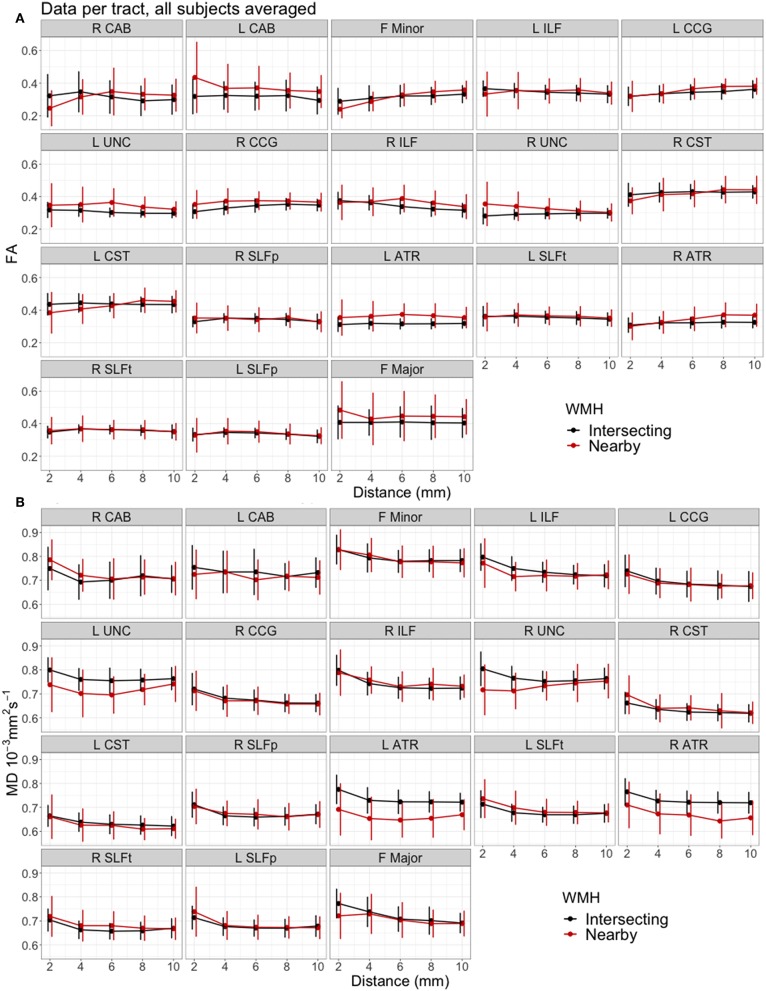
Mean water diffusion changes in tract-NAWM with distance to intersecting (tract-WMH) and nearby WMH, for each individual tract. **(A)** Fractional anisotropy (FA), **(B)** mean diffusivity (MD). Error bars show standard deviation. Tracts are ordered by median % WMH overlap in tract, as in Figure 2. L, left; R, right; F, forceps; ATR, anterior thalamic radiation; CCG, cingulate cingulum; CAB, cingulum angular bundle; CST, corticospinal tract; ILF, inferior longitudinal fasciculus; SLFp, parietal superior longitudinal fasciculus; SLFt, temporal superior longitudinal fasciculus; UNC, uncinate fasciculus.

Figure 4 shows the measured FA and MD averaged for all participants, plotted against distance from intersecting and nearby WMH for each individual tract. The pattern of diffusion changes with distance varied slightly between tracts. Tracts in Figure 4 are ordered by increasing median % WMH overlap in tract, as per Figure 2. There was no observable trend in the patterns of changes according to the %WMH in the tract.

Table 4 shows the results from the models. A significant increase of FA with Log(distance) was observed for F Minor, bilateral CCG and right CST, and significant decrease in right ILF. Additionally, FA was significantly lower for nearby WMH type in the F Minor, left CST and right ATR, and significantly higher for the F Major, left ATR, and bilateral CAB, CCG, and UNC.

**Table 4:** Results from the repeated-measures linear mixed model analysis for the analysis of water diffusion in tract-WMH and nearby-WMH contours for individual tracts.

**Predictor**	**Log(distance)**					**WMH “nearby”**					**Log(distance): WMH “nearby”**				
**Tract**	**Estimate**	**Std. error**	**Df.res**	***F***	***p***	**Estimate**	**Std. error**	**Df.res**	***F***	***p***	**Estimate**	**Std. error**	**Df.res**	***F***	***p***
**Outcome**	**FA**														
F Major	−0.005	0.011	33.9	0.2	0.681	0.039	0.011	256.2	13.0	0.000					
F Minor	0.029	0.007	79.6	15.8	0.000	−0.085	0.015	325.7	32.9	0.000	0.049	0.008	325.3	33.8	<0.001
L ATR	0.004	0.007	44.2	0.3	0.595	0.045	0.005	346.4	72.4	0.000					
L CAB	−0.030	0.020	32.2	2.3	0.139	0.042	0.013	156.0	10.9	0.001					
L CCG	0.035	0.006	40.9	31.6	0.000	0.015	0.005	326.3	8.8	0.003					
L CST	−0.003	0.009	70.2	0.1	0.716	−0.099	0.019	352.6	27.4	0.000	0.053	0.0102	349.4	26.6	<0.001
L ILF	−0.014	0.007	43.0	4.0	0.053	0.005	0.006	281.5	0.8	0.361					
L SLFp	−0.006	0.006	40.4	0.8	0.371	0.004	0.004	306.1	0.7	0.417					
L SLFt	−0.008	0.006	42.7	1.7	0.198	0.006	0.004	325.0	2.7	0.104					
L UNC	−0.017	0.007	40.0	5.3	0.026	0.038	0.006	305.1	46.4	0.000					
R ATR	0.010	0.007	75.1	1.9	0.171	−0.045	0.016	351.8	7.3	0.007	0.039	0.009	347.2	18.3	<0.001
R CAB	0.005	0.015	36.1	0.1	0.728	0.046	0.013	212.8	12.2	0.001					
R CCG	0.018	0.005	39.3	12.4	0.001	0.036	0.005	306.3	44.1	0.000					
R CST	0.025	0.007	48.6	12.4	0.001	−0.001	0.005	372.4	0.0	0.852					
R ILF	−0.029	0.006	43.4	25.6	0.000	0.019	0.006	292.7	11.5	0.001					
R SLFp	−0.002	0.005	40.4	0.2	0.661	0.002	0.005	318.4	0.3	0.608					
R SLFt	0.000	0.005	45.5	0.0	0.976	0.004	0.004	344.6	1.1	0.300					
R UNC	0.012	0.008	66.2	2.1	0.150	0.120	0.019	312.8	41.1	0.000	−0.050	0.010	313.3	25.0	<0.001
**Outcome**	**MD**														
F Major	−0.044	0.006	33.8	48.9	0.000	−0.010	0.006	258.7	2.7	0.099					
F Minor	−0.034	0.004	38.3	82.7	0.000	0.005	0.004	310.5	1.3	0.262					
L ATR	−0.024	0.006	43.5	15.9	0.000	−0.071	0.005	345.0	168.0	0.000					
L CAB	−0.018	0.012	30.4	2.4	0.134	−0.031	0.011	175.6	7.1	0.008					
L CCG	−0.036	0.006	40.6	36.8	0.000	−0.004	0.005	321.1	0.7	0.398					
L CST	−0.027	0.004	46.1	49.7	0.000	−0.013	0.004	336.6	13.1	0.000					
L ILF	−0.041	0.006	42.9	53.3	0.000	−0.011	0.005	279.6	5.8	0.016					
L SLFp	−0.028	0.005	40.4	35.7	0.000	0.004	0.003	303.6	1.2	0.283					
L SLFt	−0.031	0.004	42.3	54.4	0.000	0.011	0.004	326.0	10.3	0.001					
L UNC	−0.013	0.006	39.4	4.9	0.033	−0.044	0.005	302.9	67.3	0.000					
R ATR	−0.029	0.005	42.4	38.1	0.000	−0.059	0.005	333.0	147.0	0.000					
R CAB	−0.030	0.010	34.5	9.9	0.003	0.002	0.010	219.2	0.0	0.859					
R CCG	−0.035	0.005	39.9	48.9	0.000	−0.012	0.005	305.1	5.9	0.016					
R CST	−0.031	0.004	47.3	71.3	0.000	0.009	0.004	375.0	6.3	0.013					
R ILF	−0.042	0.006	43.8	52.5	0.000	0.010	0.004	285.8	4.5	0.034					
R SLFp	−0.025	0.005	40.5	27.2	0.000	0.007	0.004	312.3	3.4	0.067					
R SLFt	−0.027	0.004	45.2	49.3	0.000	0.013	0.003	340.9	16.1	0.000					
R UNC	−0.030	0.008	73.8	13.5	0.000	−0.144	0.020	309.3	50.5	0.000	0.063	0.011	313.1	33.8	<0.001

*WMH, white matter hyperintensity; FA, fractional anisotropy; MD, mean diffusivity in (10^−3^ mm^2^/s); L, left; R, right; F, forceps; ATR, anterior thalamic radiation; CCG, cingulate cingulum; CAB, cingulum angular bundle; CST, corticospinal tract; ILF, inferior longitudinal fasciculus; SLFp, parietal superior longitudinal fasciculus; SLFt, temporal superior longitudinal fasciculus; UNC, uncinate fasciculus*.

A significant decrease of MD with Log(distance) was observed for all tracts, except for the left CAB and left UNC. Additionally, a significantly lower MD for the contours of nearby vs. intersecting WMH type was observed for the bilateral ATR, left CAB, left CST and bilateral UNC, while MD was significantly higher for nearby vs. intersecting WMH type in bilateral SLFt.

The interaction term log(distance):WMH type was significant and improved the model in F Minor, left CST, right ATR and right UNC for FA, and in right UNC for MD.

## Discussion

This is the largest study to date quantitatively describing changes in the microstructure of main brain WM tracts with distance from intersecting WMH. We observed a pattern of decreasing abnormalities as we moved further away from the intersecting WMH and along the WM tract. A similar distance pattern of abnormalities was observed in WM tract tissue at specific distances away from nearby WMH that were not directly connected to the tract. This indicates that microstructural tissue changes in WM tracts spread beyond the visible damage of the WMH, and also suggests that WMH have similar effects on tracts whether they are intersecting or nearby the tract.

A few studies of brain aging have specifically analyzed the structure of WM tracts intersecting with WMH (14, 34–37). They generally found the largest rates of WMH overlap in the anterior thalamic radiation, inferior longitudinal fasciculus, forceps major, posterior thalamic radiation and the inferior fronto-occipital fasciculus. We found similar rates of overlap for the forceps major and anterior thalamic radiation, but also observed large overlap in other tracts, such as in the parietal and temporal superior longitudinal fasciculi (median 2.0–2.7%, with several subjects with >25%), and with WMH affecting tracts in either brain hemisphere to a similar level (Figure 2). Differences in the overlap patterns with previous studies could be due to different tractography or registration methods employed, or by the different age ranges of the samples, as the prevalence of WMH is highly dependent on age (38). All the participants in our study were born in the same year, and therefore very close in age at time of MRI scanning, minimizing the effect of age on other results. However, we aimed to include participants to represent the whole range of WMH burden (Fazekas scores). As expected, the distribution of Fazekas scores in Figure 2 shows that those with lower Fazekas scores tend to have less WMH overlap in all tracts.

The incidence of WMH in specific tracts was not reported in previous studies, however the patterns of overlap that we observe agree with previous analyses on the distribution of WMH (39, 40). The CST had the highest percentage of participants with a WMH in this tract (>92%; Table 1), with a median WMH volume of 1.7%. The high incidence may be explained by the proximity of this tract to the lateral ventricles, and the common occurrence of periventricular WMH (41). Similarly, due to the typical pattern of distribution of WMH, the percentages of participants with a WMH in the forceps minor and the uncinate fasciculus, both tracts prominently passing through periventricular areas, were high (75–82.7%), but with smaller WMH volumes (median 0.5–1.3%). Additionally, we observed that the cingulum angular bundle was least often affected by WMH (46.2–50%). This might be explained by the smaller size of this tract, or because its section within the periventricular area is mostly located medially of the posterior part of the lateral ventricle, whereas periventricular WMH are mainly observed lateral to the anterior or posterior ends of the lateral ventricles (41). It can also be noted from Figure 2, that most participants with a low total Fazekas score did not have WMH overlapping with the cingulum angular bundle, while other tracts do overlap with WMH at all Fazekas scores, suggesting that the cingulum angular bundle is affected only in persons with more severe WMH.

Regarding the microstructural quality of the WM, for the majority of the 18 WM tracts assessed here, we observed the expected pattern with higher MD and lower FA in tract-WMH. Both parameters then normalize as we move away from the visible damage, with FA significantly increasing and MD significantly decreasing as we get 2–10 mm away from the visible WMH. Such microstructural abnormalities may be caused by a variety of microvascular dysfunctions, resulting in interstitial edema, inflammation, ischemia and damage to the myelin sheath of WM tracts, ultimately leading to visible WMH (42).

These results agree with a similar analysis of tracts crossing through WMH (14), although they are not entirely in line with previous studies conducted by our group of the whole NAWM (13, 43). In our previous analysis, we *qualitatively* observed a similar distance pattern for whole-brain NAWM up to and including 4 mm distance from WMH edge for FA and 8 mm from WMH edge for MD, but at further distances the abnormalities in the NAWM apparently increased (with reducing FA and increasing MD). This might be explained by the fact that previously we looked at whole-brain NAWM rather than tract specific regions, hence may have found a “location bias” where the WMH contours extended across WM tracts (13). The current study did not suffer from location bias because water diffusion was measured in NAWM contours specifically in the same tract.

Another location effect to be considered is that WMH typically appear in areas of the brain where there is complex white matter fiber architecture (e.g., fiber bundle crossing, bending or kissing), such as periventricular regions. This could in turn affect values of the water diffusion metrics measured in a specific tract (44), and hence the changes observed in contours moving away from the WMH could be caused by the changing fiber architecture, rather than by the WMH. This effect could be mitigated in an individual tract by measuring also the water diffusion parameters in a non-WMH affected tract. However, FA and MD values are relatively specific to each tract system (45), and water diffusion parameters measured along a tract show very specific patterns (46). Hence this correction could only be performed between equivalent contralateral white matter tracts. WMH tend to be quite symmetrical between brain hemispheres (41, 47), even when there is an asymmetry in potential risk factors, such as blood supply (48), and there are typically insufficient equivalent areas of each tract affected by WMH in one hemisphere, but unaffected in the contralateral hemisphere, to perform such a correction. Nevertheless, we performed a supplementary analysis of FA and MD measured in a tract with complex fiber architecture and compared the values measured in equivalent areas in participants with and without WMH. We found that any residual effects of fiber architecture in tract sections equivalent to those surrounding a WMH would not fully account for the changes we observed around a WMH (see Supplement 1).

We also measured the diffusion characteristics in areas of tracts that were nearby, but not directly connected to a WMH, to study the effects of these nearby WMH on the tracts. It has been suggested that, from a biological perspective, WMH in a WM tract would influence the tissue integrity along the rest of the tract more strongly than a nearby (non-directly connected) WMH since the interstitial fluid changes and secondary degenerative processes could theoretically propagate more easily along the tract than in adjacent non-tract tissue where cell processes are less well aligned (12, 37). This interpretation was supported by Reginold et al. (14) who analyzed diffusion changes in WM tracts with distance from WMH and reported that CST tracts traversing WMH have worse diffusion characteristics (MD, axial, and radial diffusivity, but not FA) at the same distance from WMH, compared to CST tracts that did not intersect but were close to a WMH, concluding that WMH may be causing abnormalities in NAWM through Wallerian-type degeneration. Our results are consistent with these findings and our plots of FA and MD for the CST in Figure 4 show similar trajectories to those in Reginold et al. (14). In particular, we also observed higher MD in areas surrounding tract-WMH compared to nearby-WMH contours (Table 4). The changes for FA in this tract were however more complex; Figure 4A shows that, for both CST, the areas of the tract nearby, but not directly connected to WMH, had lower FA than areas at similar distance surrounding an intersecting WMH, up to a distance approximately of 6 mm, while at further distances FA was higher for “nearby” contours. Low FA can indicate axonal injury and myelin loss, hence the lower values of FA in the nearby-WMH contours, up to 6 mm, suggest lower quality of the WM in these areas, compared to the intersecting-WMH contours. A similar effect is also observed in other tracts, such as the right CAB, F Minor and the R ATR, indicating that in these tracts the damage is more localized around the nearby-WMH. For both WMH types the FA increased with distance from WMH with a higher gradient for nearby-WMH contours, with FA trends for both WMH types crossing over at 4–6 mm suggesting a faster normalization for nearby-WMH contours in some tracts.

Our analysis including all 18 tracts in the model also supports previous studies (14). Our full model including data from all tracts in Figure 3 shows that MD was consistently lower, while FA was higher in nearby-WMH contours compared with intersecting WMH contours. For both WMH types, MD decreased while FA increased with distance from WMH. The estimated differences observed for both type of WMH were small but significant for both FA and MD. Our results may support the hypothesis that age-related WM damage propagates further along tract axons, potentially through enabling propagation of interstitial fluid or through Wallerian-type degeneration, as previous studies suggested. However, the larger accumulation of damage around the nearby WMH (lower FA) in some tracts, may suggest that different mechanisms of tissue damage propagation also play a role. For example, age-related WMH are associated with other small vessel disease (SVD) features, such as widening of perivascular spaces (1, 49, 50), that reflect microvascular dysfunction including abnormal blood-brain barrier leakage, impaired vasoreactivity and impaired pulsatility. Other SVD studies also found that MD is more abnormal than FA in NAWM adjacent to WMH (51, 52), suggesting other potential channels for the propagation of tissue damage, inducing FA and MD changes, independently of the direct connections of WM tracts. As shown in Figure 4, the effects of WMH in WM tracts are variable across the brain. A detailed study of this variation across tracts and locations in the brain could help pinpointing the processes underlying the microstructural changes detected and their effects on cognitive and physical function.

Water diffusion abnormalities within the NAWM are suggestive of tissue damage not yet visible on a lesion level. It is, however, unclear whether the WMH are the cause of the NAWM damage, or whether both are part of the same continuum of tissue damage (53). These water diffusion abnormalities are a precursor to lesion extension or development within the NAWM (53–56), which reflect the general association between higher WMH load, worse NAWM MD and more WMH growth. As lesions are associated with cognitive abnormalities (7, 8, 57), such lesion development in NAWM areas suggests that pre-lesional NAWM water diffusion abnormalities may already play a role in cognitive functioning (58, 59). Previous studies have found that the rate of WMH growth was heterogeneous, occurring more rapidly within some association and projection tracts compared to other white matter regions (39). A longitudinal study of this growth at the tract level, and with respect to the location of tract-WMH and nearby WMH induced changes in the tract-NAWM, could help explain the patterns of WMH progression.

This study has some limitations. Firstly, it has a relatively small sample, mainly for the individual tract comparisons where there were few cases with WMH in some tracts. However, the current study is the largest to date looking at specific locations of NAWM in WM tracts with distance from the WMH and its results will be used as motivation for investigating WMH-tract interactions in larger samples. The current study can be further extended to focus on relating water diffusion in tract-WMH and tract-NAWM to premorbid cognition, current cognitive functioning, and on the development of water diffusion abnormalities in tract-WMH and tract-NAWM over time. Secondly, the closer spatial contours were not available for every tract as some nearby WMH were too far away for a 2- or 4-mm contour to cross with the tract. This means that there were less data contributing to the calculation of water diffusion for 2 and 4 mm and their measurements might be less accurate than for the tract-WMH contours. However, we used linear mixed models for our analysis, which are less sensitive to missing data and allow the inclusion of location data for all tracts without introducing a bias. Thirdly, water diffusion metrics need to be interpreted with care in regions with complex fiber configurations, such as crossing or bending fibers, which might confound the measured changes in water diffusion metrics. The imaging parameters and methodology used for analyzing the diffusion data might also have a significant effect in such a fine-grained analysis, therefore the patterns of change with distance will need to be corroborated in larger studies.

In conclusion, microstructural changes in tract-NAWM became less pronounced along the tract and further away from the tract-WMH, with a comparable distance pattern away from the nearby (not intersecting) WMH. The observed differences in tissue microstructure between the WM tract areas surrounding intersecting WMH and those surrounding nearby non-intersecting WMH suggest that WM degenerative processes in SVD may propagate further along the tract for intersecting WMH. In some areas of the brain there is a larger and more localized accumulation of axonal damage in tract tissue nearby a non-connected WMH, suggesting that different channels of accumulation of the damage need to be explored. The tissue damage in WM tracts observed beyond visible lesions may contribute to cortical disconnection and changes in cognitive functioning. Our future efforts are aimed at elucidating the relationship between microstructural changes of nearby and intersecting-WMH, tract-NAWM and cognitive functioning at older ages, and at the development of tissue changes in tract-NAWM over time.

## Data Availability

The datasets generated for this study are available on request to the corresponding author.

## Ethics Statement

Written informed consent was obtained from all participants under protocols approved by the National Health Service Ethics Committees.

## Author Contributions

SM and RM conducted the MRI tractography, data processing and statistical analyses, and drafted the initial manuscript. FC assisted with data statistical analysis and interpretation. MV conducted the tissue segmentation analysis. MB designed the MRI protocol and assisted with manuscript. JS, ID, and JW designed and conceived the LBC1936 study and assisted with manuscript. JW performed WMH visual scores and assisted with data interpretation.

### Conflict of Interest Statement

The authors declare that the research was conducted in the absence of any commercial or financial relationships that could be construed as a potential conflict of interest.
