# Supplementary material for: Spatial Gradient of Microstructural Changes in Normal-Appearing White Matter in Tracts Affected by White Matter Hyperintensities in Older Age
Source: Front Neurol. 2019 Jul 25;10:784. doi: 10.3389/fneur.2019.00784 (PMC6673707; doi:10.3389/fneur.2019.00784)
Supplement: Supplementary file 1 [file Presentation_1.pdf]

Supplement 1: Analysis of potential effects of tract architecture in FA and MD

Diffusion parameters measured in white matter hyperintensities (WMH) contours located on the arch, or other area of complex architecture, within a white matter tract could be affected by fibre complexity and the changing fibre tract orientation in the tract (Vos et al., 2012).

To discern real effects of WMH from potential effects of tract architecture in the fractional anisotropy (FA) FA and mean diffusion (MD) measured along contiguous sections of a tract, we selected a particular WMH located in the arch of the right superior longitudinal fasciculus (R SLFt) of one study participant, as shown in Figure 1A, with 2mm contours around this WMH moving across areas with different fibre orientation and complexity (Catani et al., 2005).

We identified eight participants from the study sample without WMH in this tract and registered non-linearly the selected WMH mask into their native space, in order to simulate a WMH in the arch of otherwise healthy R SLFt. The intersections between the registered WMH mask and the native R SLFt were identified, and contours of 2mm thickness were created in the tract's normal-appearing white matter (tract-NAWM) around this simulated WMH. Figure 1B shows examples of the simulated WMH and the contours surrounding it in the native space of three WMH-free R SLFt cases.

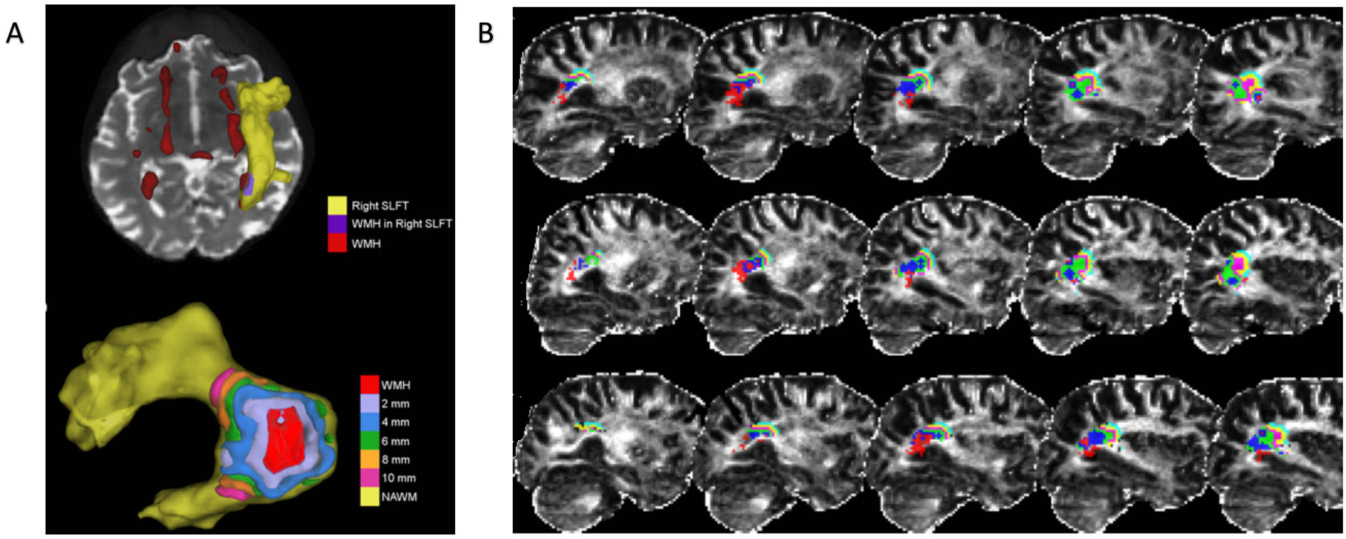

Figure1. WMH in the arch of the right superior longitudinal fasciculus (A) was registered into the diffusion maps of participants without WMH in this tract. (B) shows the registered WMH (red) and the 2mm contours created around it for three participants.

FA and MD were measured in the simulated WMH and contour areas and compared to the FA and MD measured in real WMH in the R SLFt for the remaining 44 participants, and their corresponding contours, see Figure 2. The data from real WMH originates from any WMH intersecting the R SLFt, independently of its location, and therefore not only from the same exact location as the simulated WMH, however it serves as a reference for a qualitative comparison with the simulated WMH data.

We did not observe any obvious trend of changes of FA with distance from the simulated WMH (Figure 2A), whereas there was a trend of increasing FA from real WMH up to 4mm (Figure 2B).

We observed a steady decrease of MD with distance from the simulated WMH (Figure 2C), although this was gradual and did not follow the logarithmic pattern of changes observed around real WMH (Figure 2D). This could potentially indicate a residual effect of complex fibre configurations in the values of MD as we measured across the fibre arch. However, this was not supported with analogous changes in FA, neither accounts for the full changes observed in areas with real WMH. Given the proximity of the simulated WMH and the closest contours to the lateral ventricle (Figure 1), partial volume averaging with CSF and potential inaccuracies during the registration could also contribute to the observed changes in MD.

In conclusion, we do not observe a clear effect of the changing fibre orientation and complexity in the values of FA as we measure along the arch of a white matter fibre. A potential latent effect of fibre orientation was observed in MD, however this did not account fully for the changes observed in the proximity of a real WMH.

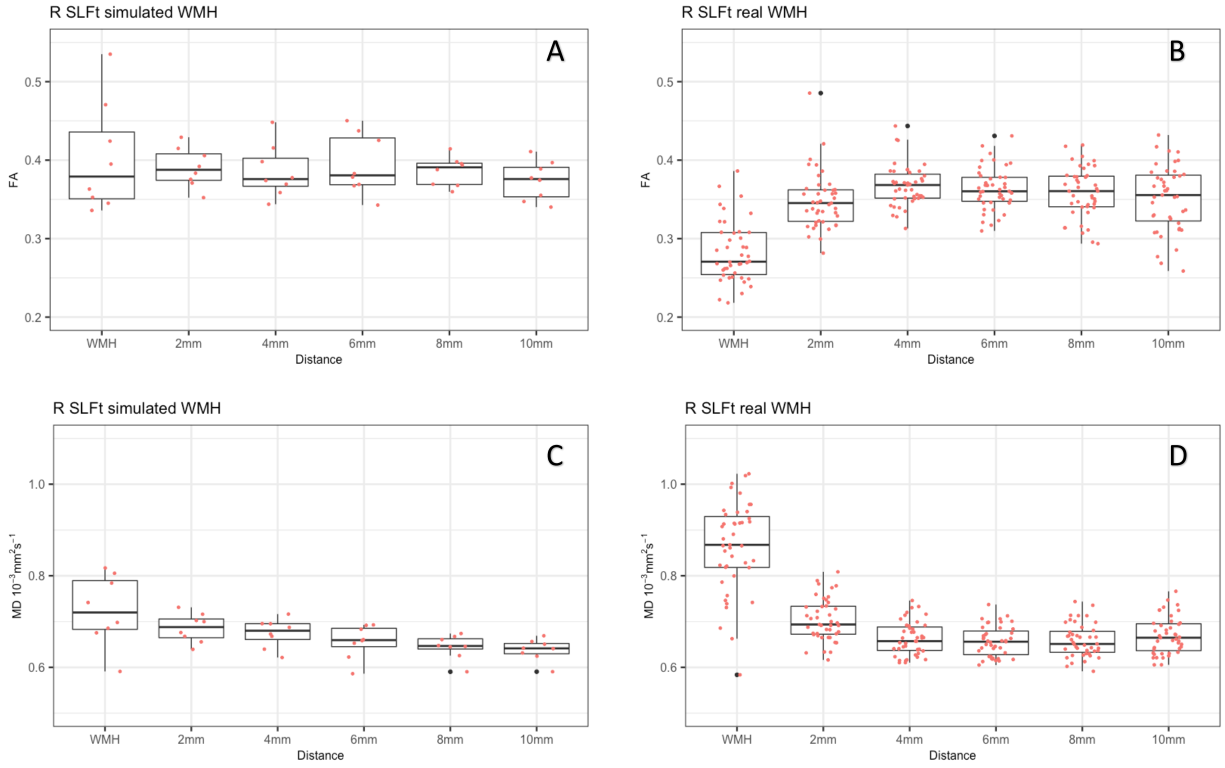

Figure2. FA (top) and MD (bottom) measured in the simulated WMH (left) and real WMH (right) and their surrounding contours. The boxes represent the lower and upper quartiles and the median measurement (thick line) for each tract, while whiskers indicate the sample minimum and maximum, excluding outliers that differ from the lower and upper quartiles by more than 1.5 times the interquartile range. The red dots represent individual data.

Catani, M., Jones, D.K., ffytche, D.H., 2005. Perisylvian language networks of the human brain. Ann. Neurol. 57, 8–16. doi:10.1002/ana.20319

Vos, S.B., Jones, D.K., Jeurissen, B., Viergever, M.A., Leemans, A., 2012. The influence of complex white matter architecture on the mean diffusivity in diffusion tensor MRI of the human brain. Neuroimage 59, 2208–2216. doi:10.1016/J.NEUROIMAGE.2011.09.086
