# Supplementary material for: Spatial Gradient of Microstructural Changes in Normal-Appearing White Matter in Tracts Affected by White Matter Hyperintensities in Older Age
Source: Front Neurol. 2019 Jul 25;10:784. doi: 10.3389/fneur.2019.00784 (PMC6673707; doi:10.3389/fneur.2019.00784)
Supplement: Supplementary file 2 [file Table_1.docx]

**Supplementary Table 1** Descriptive statistics of the percentage of tract volume intersecting WMH

| **% WMH** | **L/R** | **Mean** | **Median** | **Min** | **Max** | **Q1** | **Q3** | **IQR** |
| --- | --- | --- | --- | --- | --- | --- | --- | --- |
| **F Major** | n/a | 5.0 | 2.8 | 0.0 | 23.3 | 0.5 | 7.2 | 6.7 |
| **F Minor** | n/a | 1.2 | 0.5 | 0.0 | 9.1 | 0.1 | 1.4 | 1.3 |
| **ATR** | L | 3.8 | 2.0 | 0.0 | 18.6 | 0.9 | 5.9 | 5.0 |
| **ATR** | R | 4.4 | 2.5 | 0.0 | 34.4 | 0.9 | 5.1 | 4.2 |
| **CCG** | L | 1.8 | 0.9 | 0.0 | 9.5 | 0.1 | 2.7 | 2.7 |
| **CCG** | R | 3.4 | 1.2 | 0.0 | 18.0 | 0.2 | 4.7 | 4.5 |
| **CAB** | L | 0.8 | 0.3 | 0.0 | 5.6 | 0.0 | 1.0 | 1.0 |
| **CAB** | R | 1.5 | 0.2 | 0.0 | 22.2 | 0.0 | 1.0 | 1.0 |
| **CST** | L | 4.6 | 1.7 | 0.0 | 37.6 | 0.3 | 7.8 | 7.5 |
| **CST** | R | 4.0 | 1.7 | 0.0 | 39.1 | 0.5 | 3.8 | 3.3 |
| **ILF** | L | 2.8 | 0.9 | 0.0 | 25.1 | 0.1 | 3.3 | 3.2 |
| **ILF** | R | 2.9 | 1.3 | 0.0 | 33.2 | 0.1 | 4.0 | 3.8 |
| **SLFp** | L | 5.8 | 2.7 | 0.0 | 27.5 | 0.5 | 9.7 | 9.2 |
| **SLFp** | R | 5.6 | 1.8 | 0.0 | 47.3 | 0.3 | 6.6 | 6.3 |
| **SLFt** | L | 5.7 | 2.6 | 0.0 | 28.3 | 0.4 | 7.1 | 6.7 |
| **SLFt** | R | 5.5 | 2.3 | 0.0 | 28.5 | 0.3 | 6.8 | 6.5 |
| **UNC** | L | 2.2 | 1.1 | 0.0 | 21.2 | 0.5 | 2.7 | 2.1 |
| **UNC** | R | 1.7 | 1.3 | 0.0 | 7.3 | 0.2 | 2.8 | 2.5 |

*WMH =white matter hyperintensity, L= left, R=right, F= forceps, ATR = anterior thalamic radiation, CCG = cingulate cingulum, CAB = cingulum angular bundle, CST = corticospinal tract, ILF = inferior longitudinal fasciculus, SLFp = parietal superior longitudinal fasciculus, SLFt = temporal superior longitudinal fasciculus UNC = uncinate fasciculus. Q1 = first quartile, Q3 = third quartile, IQR = interquartile range. Note: ‘Max’ values are inclusive of outliers.*
