# Supplementary figures and images for: Spatial Gradient of Microstructural Changes in Normal-Appearing White Matter in Tracts Affected by White Matter Hyperintensities in Older Age
Source: Front Neurol. 2019 Jul 25;10:784. doi: 10.3389/fneur.2019.00784 (PMC6673707; doi:10.3389/fneur.2019.00784)

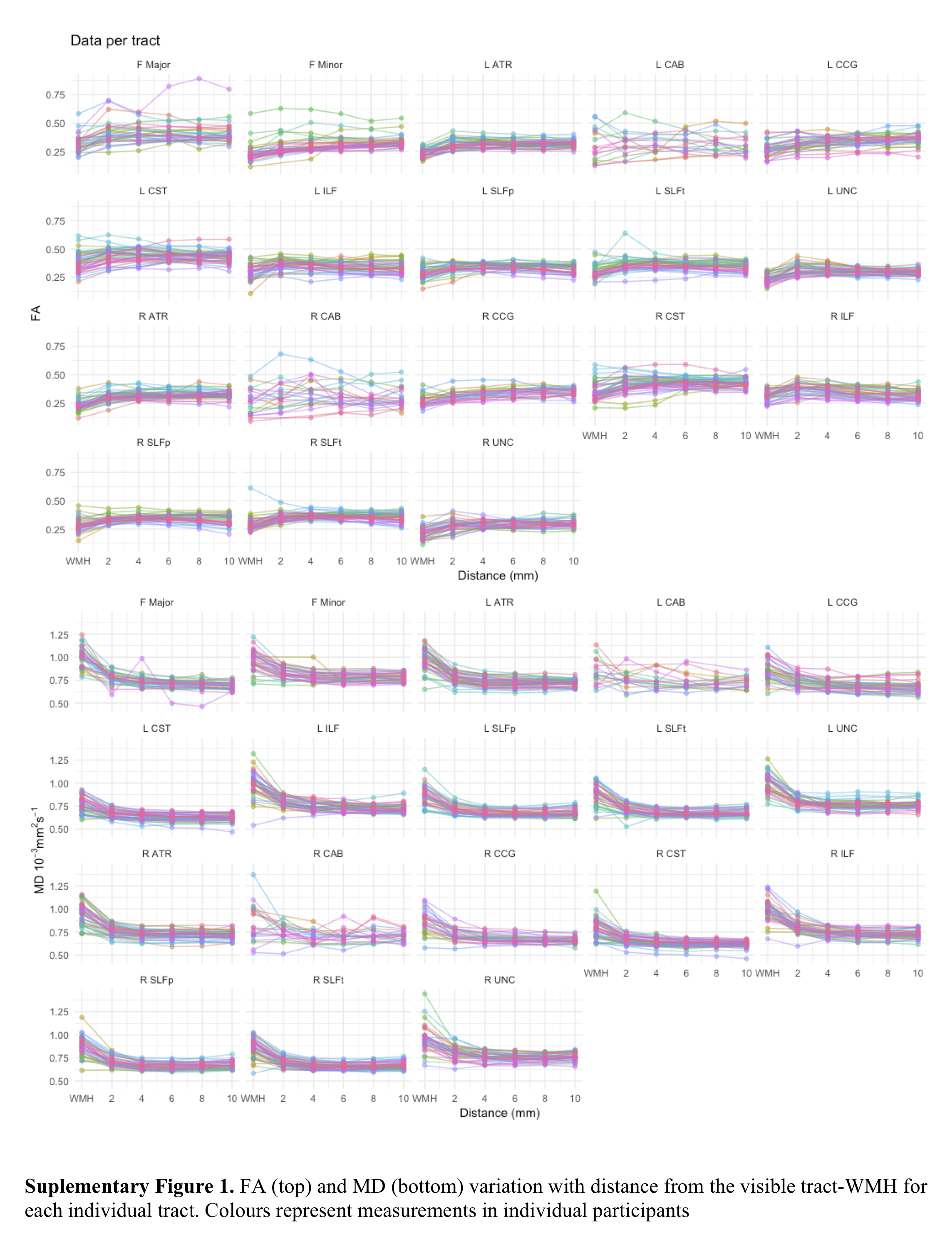

Supplement: Supplementary file 3 [file Image_1.TIFF]
